# Second Clinical Report on Continued Fever, Based on an Analysis of Forty-Eight Cases

**Published:** 1851-10

**Authors:** Austin Flint

**Affiliations:** Prof. of the Principles and Practice of Medicine, and Clinical Medicine, in the University of Buffalo


ART. V.—*Second Clinical Report on Continued Fever, based on an analysis of forty-eight cases.* By AUSTIN FLINT, M. D., Prof. of the Principles and Practice of Medicine, and Clinical Medicine, in the University of Buffalo.

(Continued from Page 237.)

SECTION SIXTH.

*Cutaneous Eruptions.* [No. for Nov., page 335.]

*Typhoid.* The characteristic eruption was present in *twelve*, of the *twenty-nine Typhoid* cases. This result differs considerably from that obtained by the first analysis. The latter developed a larger proportion of cases in which this symptom was present, viz., in *twenty-three,* of *thirty* cases; the ratio, thus, being over *two-thirds*, while in the present collection it is less than *one-half.* Of the whole number of cases in both collections, viz., *fifty-nine,* it was present in *thirty-five.* I can offer no explanation of the above disparity. Careful examinations for the eruption were made in every instance, so that it is hardly possible that it could have been overlooked, and in all the cases in which it is not stated to have been present, the fact of its absence is distinctly noted in the record of each day.

The eruption exhibited the distinctive characters of that belonging to the *Typhoid* type of fever, mentioned in the first Report, in all the cases in which it was present.

The rose spots were not abundant save in *two* cases. They were confined to the abdomen and chest in every case but *one*, in the latter case extending over the upper and lower extremities.

The period in the disease at which the eruption appeared, dating from the commencement of the access, or of the febrile career, is not accurately determinable in many cases, owing to the histories being defective as to the duration of illness prior to the patients coming under observation. The period after admission at which the eruption was observed, is generally stated, but in nearly every instance, the disease had existed for a greater or less length of time before the patients entered the hospital. The facts recorded with respect to this point, are presented in the following table, which embraces also the duration of the eruption, dating from the time of admission into hospital:

ERUPTION IN TWELVE TYPHOID CASES.

| APPEARANCE OF ERUPTION. | PERIOD WHEN ERUPTION WAS MUCH FADED, OR HAD DISAPPEARED. |
|---|---|
| Case 1. 8th day after entrance. | 14th day. |
| " 2. 5th day do. Had been ill ten days. | 12th day. |
| " 3. 2d day after entrance. Previous duration of illness not noted. | 6th day. |
| " 4. Existed when admitted. Had been ill about five days. | Not noted. |
| " 5. 5th day after entrance. Previous duration of illness not noted. | 10th day. |
| " 6. 2d day. Previous duration of illness not noted. | 6th day. |
| " 7. Existed when admitted. Had been ill ten days. | 7th day. |
| " 8. Existed when admitted. Had been ill fourteen days. | 4th day. |
| " 9. Existed when admitted. Had been ill ten days and confined to bed four days. | Not noted. |
| " 10. Existed when admitted. Had been ill several days, but rode thirty miles day before, and walked to hospital. | Not noted. |
| " 11. Existed when admitted. Previous duration of illness not noted. | 8th day. |
| " 12. Existed when admitted. Had been ill several days. | 6th day. |

The eruption in all the cases faded, from day to day, before finally disappearing.

*Typhus.* The characteristic eruption of *Typhus* was present in *eight*, of the *ten* cases of this type. An eruption was thus present in a much larger proportion of cases than in the cases of *Typhoid.* The result does not present so much disparity when compared with that of the former analysis, as in the *Typhoid* cases. Of *thirteen* cases in the first collection, an eruption existed in all. In the *Typhus* cases of both collections, viz., *twenty-three,* it was present in *twenty-one.* So far as these observations go, they show the occurrence of an eruption in a very large majority of the cases of this type of Continued Fever.

In *six* of the *eight* cases, the eruption was copious, and its abundance in the *two* remaining cases is not stated.

In *six* of the cases the eruption was not limited to the trunk, but extended over the upper and lower extremities. In the *two* remaining cases the histories do not state as to this point.

The eruption presented the distinctive characters of *Typhus*, mentioned in the first Report, with the following exceptions: In *one* case rose spots were intermingled with the maculæ; and in *one* case the redness partially disappeared on pressure. A few vesicles were intermingled with the maculæ in *one* case.

The facts pertaining to the first appearance, and the duration of the eruption in the *Typhus* cases, are presented in the following table:

ERUPTION IN EIGHT CASES OF TYPHUS.

| APPEARANCE OF ERURPION. | PERIOD WHEN ERUPTION HAD MUCH FADED, OR DISAPPEARED. |
|---|---|
| Case 1. Present when admitted. Had been ill four days, but had not kept the bed. | Not stated. |
| " 2. 8th day. | 11th day. |
| " 3. Present when admitted. Had been ill six days, and taken to bed day before admission. | Not stated. |
| " 4. 2d day after admission, and 2d day after taking to bed. | 5th day. |
| " 5. 4th day after admission, and 4th day after taking to bed. | 9th day. |
| " 6. Present when admitted. Previons duration of illness not stated, | Not stated. |
| " 7. 2d day after admission, and 2d day after taking to bed. | 7th day. |
| " 8. 3d day after taking to bed. | 11th day. |

Of the cases of *Doubtful type*, an eruption appeared in *one* case only. The characters of the eruption in this case were such as to render it difficult to decide whether it resembled most that of *Typhus*, or *Typhoid*. It appeared on the *seventh* day after taking to the bed, and the *tenth* after illness commenced. The appearances are thus described in the history: "The eruption is pretty copious over the chest and abdomen, and extends, but sparsely, over the upper extremities. It is not precisely the eruption of either *Typhus* or *Typhoid*, but approaches nearer the latter. The spots are of a duller red color, are smaller, and not so distinctly elevated, or papular. The redness disappears on pressure, but not in so marked a degree, as is usually the case in *Typhoid*. After repeated examinations, and considerable hesitancy, I think the eruption is rather like that of *Typhoid*, than that of *Typhus*."

Afterward, the following record was made: — "The eruption evidently is of two kinds, the *Typhus* and *Typhoid*. *It extends over the face*."

On the next day it is stated "the eruption to-day presents more the appearance of *Typhus*."

With respect to the existence of an eruption in the fatal cases, the facts are as follows: — Of the *seven* fatal cases of *Typhoid*, in *three* an eruption existed, and in *four* it was absent. Of the *four* fatal cases of *Typhus*, in *two*, an eruption was present, and in *two* it was absent, the latter being the only cases of this type in which no eruption existed. These results accord very closely with those developed by the first analysis, limiting the attention to the *Typhoid* cases, which is manifestly proper, in so far as the former collection is concerned, inasmuch as in *all* the *Typhus* cases, in that collection, an eruption was present. In the *six* fatal *Typhoid* cases among those before analysed, an eruption was present in precisely one half.

A *petechial* eruption was present in *one* case, a case of the *Typhoid* type. It appeared on the sixth day after admission, and remained for several days. The ecchymoses were numerous over the abdomen and chest, being intermingled with a sparse rose eruption. The patient at the same time had hæmorrhage from the gums. The case ended in recovery.

Sudamina were not usually sought for, and the histories are therefore deficient in information respecting this species of eruption. It is only noted in the history of a single case — a case of *Typhoid*, concurring with copious perspiration. Had sudamina been prominent in any other case, it would hardly have escaped observation. I feel sure that this symptom could not have been present in any of the cases, with the exception just mentioned, save in so obscure a degree as not to attract attention.

### SECTION SEVENTH.

*Symptoms Referable to the Respiratory Apparatus. Cough. Expectoration, Pain in Chest. Pneumonitis. Aberrations of Respiratory Movements. Epistaxis. Singultus.* [No. for November, page 338.]

*Cough. Typhoid.* — The presence of this symptom is noted in the histories of *twenty-two Typhoid* cases. Of the remaining *seven* cases of this type, its absence is noted in *three*, and nothing is stated with regard to it in *four*.

These results are not what would have been anticipated in view of those contained in the former Report. The first analysis gave, of *thirty Typhoid* cases, the presence of cough in only *ten*. Here is one of the most striking points of disparity developed by the two analyses. What is the inference? Assuming that equal care was taken in observing for this symptom, it would follow that in the two collections, which it will be observed embrace about an equal number of cases, (the one 30, and the other 29,) is exhibited a striking difference as respects liability to the morbid condition upon which the cough incident to Continued Fever depends. It is certain that the disease does manifest a development, unusual either in degree, or character, o particular phenomena, at different periods, and in different places; and the occurrence of cough in a proportion of cases so much larger in the second, than in the first collection, would appear to afford an illustration of variableness as respects this symptom. The greater preponderance of the symptom may have been owing to circumstances appertaining to the season, or to the hospital at the time the cases occurred, or it may denote special tendencies inherent in the disease itself at that time. I am unable to state which of these explanations is the correct one. It is to be considered, however, that, possibly, the disparity may not have been so great as appears from the results of the analyses. In most of the cases, as will presently be seen, the cough was slight. The fact of an occasional act of coughing, may have escaped attention in some of the cases first analyzed, and, indeed, it is remarked in the first Report, that this may have been the case. It is obvious that the symptom is one which, in many instances possessing no prominence, might fail to be observed unless special pains were taken to ascertain whether it existed, or not; and the statements of the patients themselves, for reasons already given, are not to be relied upon. I can not affirm that the results are not to be accepted with the qualification involved in this consideration; but that the disparity is not in that way fully explicable, is rendered probable by the fact the two analyses, as respects this symptom, in the *Typhus* cases, will presently be found to develop results nearly uniform. In view of this fact, the conclusion seems to my own mind a legitimate one, that, owing to causes which I will not undertake to assign, cough occurred much more frequently in the cases upon which this Report is based, than in those before analyzed.

The cough, in all except *two* cases, was either *slight,* or *moderate* in degree. In the *two* cases just referred to, it was so inconsiderable as to constitute a prominent symptom.

*Pneumonitis* existed in seven cases. This is evident in *three* of the seven cases, by the physical signs, as well as rational symptoms, which are recorded. In the *four* remaining cases, the existence of this complication is denoted by the symptoms alone, viz., *rusty* and muco-purulent expectoration, accelerated respiration, dilation of the alæ nasi, etc. Physical exploration was probably not omitted in any of these cases, but the signs are not noted in the histories. The occurrence of pneumonitis is noted in only *two* of the *thirty Typhoid* cases in the first collection.

In *four* of the *seven* cases in which pneumonitis existed, the disease proved fatal. In other words, of the *seven* fatal *Typhoid* cases, *three* had this complication. Hence, it may reasonably be inferred that the danger of a fatal termination is considerably enhanced by this complication.

The cough was slight in *six* of the *seven* cases in which pneumonitis existed. This is worthy of notice, showing that the absence of this complication is not to be predicated on the fact that cough is not prominent as a symptom.

In the *two* cases ending in recovery, in which pneumonitis existed, convalescence was apparently thereby retarded, and prolonged.

*Pain in the chest* is noted in *two* only of the cases characterized by pneumonitis. It is noted in a few other cases, viz., in *five.* In *two* of these cases, the seat of the pain was *beneath the sternum;* in *one*, the situation is not stated; in *one, beneath the shoulders,* on coughing; and in *one, on both sides.*

As respects *Expectoration,* save in the cases complicated with pneumonitis, the histories are deficient in information. This could not have been prominent, as a symptom, with the exceptions just stated. Sibilant and sonorous rales are noted in the histories of several cases, but, in general, the presence or absence of auscultatory signs were not recorded, except in some of the cases which were characterized by the occurrence of pneumonitis.

*Typhus.* Of the *ten Typhus* cases, cough was present in *seven.* The first analysis gave, of *thirteen Typhus* cases, the presence of this symptom in *eleven.* These results do not exhibit great disparity, and it will be observed that, the first collection exhibits a larger proportion of cases in which this symptom was observed, the reverse of what was developed by comparing, in this respect, the *Typhoid* cases.

The cough was *slight* in *five* of the *seven* cases, and was considerable in the *two* remaining cases.

As regards the morbid condition, or conditions, irrespective of pneumonitis, (which occurred in a small proportion of cases,) on which the cough incident to Continued Fever depends, we may suppose that it is due to a slight catarrhal affection of the mucous membrane, possibly, in some instances, amounting to a very mild grade of bronchitis; or, what is perhaps more probable, that it arises from capillary congestion of the mucous membrane resembling that which so uniformly affects the skin. Louis says, with reference to this symptom in *Typhoid* fever, that from the acts of coughing being commonly slight, and rare in occurrence, he should have frequently failed to observe it, had he limited himself to taking notes merely of what he himself saw. He adds, that in some instances it appeared not to be a *bronchial* cough, but occurred for the purpose of removing mucus from the pharynx.

These remarks are applicable to the cases I have observed. In *two* cases, the presence of *pneumonitis* is evident, both from the signs and symptoms recorded; and in *one* case, the existence of this complication is rendered probable by the symptoms. In *two* of the above cases, the disease was fatal. In other words, of the *four* fatal *Typhus* cases, *two* were characterized by the presence of pneumonitis. In the first collection of *Typhus* cases, the proportionate number of instances in which this complication existed, was larger, the relation in the two conditions being as 5 — 13 is to 3 — 10.

*Pain in the chest* is noted but in *one* case, in that case situated in the left side, and beneath the sternum. Pneumonitis existed in that case.

Of the cases of *Doubtful type*, the histories are defective as respects cough, in *two*. In the remaining *seven* cases, cough was present in *all save one*. It was slight, except in *two* cases, in which the presence of pneumonitis is declared by the symptoms and by physical signs.

*Aberrations of Respiration.* The respirations were generally enumerated frequently during the progress of the disease. In this respect the histories are more full than in the first collection, in which it is simply stated that the respirations were either increased, or normal.

*Increased Frequency of Respirations. Typhoid.* Excluding the cases in which pneumonitis existed, the respirations were not much increased in frequency, save in *three* cases. In these *three* cases, respectively, the maximum of frequency was, 40, 32, and 32 per minute; and the mean 35 1-7, 32, and 29 per minute. Inasmuch as the histories do not contain notes of physical signs, I cannot declare positively that in these cases pneumonitis may not have been present, although, aside from the respirations, the rational indications of this complication do not appear to have existed. Of the remainder of the *Typhoid* cases, excluding those characterized by the presence of pneumonitis, in *ten*, the histories of which are pretty complete as respects the enumeration of the respiratory acts, the average number, per minute, was not far from 21½ — a moderate increase of the healthy mean. Of the ten cases furnishing this average, in some, the respirations were not at all increased. The averages in the cases respectively, are as follows:

| | | | | |
|---|---|---|---|---|
| 26, | 18, | 18, | 20, | 26, |
| 16, | 24, | 21, | 16, | 27. |

Fractions are omitted in this list. There was considerable variation frequently on different days during the career of the fever. This is shown by the following table, which exhibits the successive enumerations in a few cases:

| Case 1. Recovered. | Case 2. Fatal. | Case 3. Recovered. | Case 4. Recovered. | Case 5. Recovered. |
|---|---|---|---|---|
| 1st day, 24, | 16, | 32, | 20, | 26, |
| 2d " 24, | 18, | 32, | 16, | 20, |
| 3d " 24, | 14, | 24, | 20, | 20, |
| 4th " 30, | 16, | 20, | 20, | 22, |
| 5th " 28, | 28, | 24, | 12, | 20, |
| 6th " 28, | | 28, | 14, | |
| 7th | | | 14, | |

In the cases of *Typhoid* which were complicated with pneumonitis, the frequency of the respirations was greater. In *five* of these cases, the history of each of which contains a series of enumerations, the maximum of frequency, respectively, was as follows: 32, 32, 36, 40, 48. The average in the cases, respectively, was as follows: 24 1-2, 23 15-17, 27 1-3, 32, 35 7-12. The mean, in these cases, is not far from 28 1-5, which, it will be observed, is higher than in the ten cases in which pneumonitis did not exist, in the latter being 21½. In *four*, of the above *five* cases, the disease was fatal. An increased frequency of respirations sufficient to give the above mean, viz., 28, is of importance as an indication of the existence of pneumonitis. It is more constantly associated with that complication than a degree of cough greater than the average, and, hence, is of more value as a diagnostic symptom. It should affect also, to some extent, the prognosis unfavorably.

*Typhus.* Excluding the cases known to have been complicated with pneumonitis, the respirations became considerably increased in frequency in *three.* The maximum of frequency in these cases, respectively, was as follows: 36, 40, 44. The mean number in the same cases, respectively, is as follows: 27, 31 1-5, 33 1-5. Of the remaining *five* cases, including the two complicated with pneumonitis, the respirations were enumerated in but *two*, and of these the mean was, in one 27, and in the other, 26 4-7.

Of the *two* cases complicated with pneumonitis, the maximum frequency was, in *one* 60, and in the other 40. The mean in the first of these cases is but 26 4-5, in consequence of the pneumonitis not becoming developed until the tenth day of the disease, and the respiration, prior to that date, not being much accelerated. In the other case, the mean was 34.

On comparison of the two types, these results show, so far as they go, that in *Typhus*, the increased frequency of the respirations is greater than in *Typhoid.* This was found to be the fact in the cases before analyzed.

It would be highly interesting to determine by a comparison of the cases, whether a notable increase in the frequency of the respirations only occurs in connection, either with pneumonitis, or some other pulmonary affection compromising, to a greater or less extent, the vesicular integrity of these organs. It is certain that when more or less solidification of the lungs takes place in the cases characterized by pneumonitis, the respirations become accelerated, and, on examining the daily records, I find that frequently the development of the pulmonic inflammation is signalized by a sudden increase in the frequency of respirations, the symptoms and signs denoting that complication appearing in the histories at the same time. For example, in one of the cases of *Typhus* in which pneumonitis became developed, the respirations numbered, successively, 24, 20, 20, 20, 20, 18, 18; and then 32, 36, 60. Now, in this case, prior to the day on which the respirations numbered 32, there had been at first slight cough, but afterward no cough; no dilation of the alæ nasi; no expectoration. On the day, however, when the respirations became increased in number, are noted, cough, dilation of the alæ nasi, rusty expectoration, pain beneath sternum, and pleuritic stitch in left side; and on the day following, dullness on percussion over the inferior part of the left chest.

A considerable increase in the number of respirations should thus lead to suspicion of the development of pneumonitis; but the question is, may not the same acceleration arise irrespective of pneumonitis, or other pulmonary complications? There are but *six* cases, as has been stated, in the present collection of those of the *Typhoid* and *Typhus* type, (*three* of each,) in which the respirations were notably increased without the evidences of pneumonitis being recorded; and in these *six* cases, inasmuch as the results of physical exploration are not embraced in the histories, I am not warranted in saying that this, or some other pulmonary complication did *not* exist, although not denoted by the symptoms which are recorded. The question, therefore, must remain unsettled.

Among the cases of *Doubtful type*, there were *two* which became complicated with pneumonitis. Of these cases, the maximum in frequency of the respiration, in *one*, was 60, and in the other, 48. The mean frequency in the former was 42 4-11, and in the latter 32.

*Diminished Frequency of Respiration.* In *two* cases, the respirations are noted once in each case as low as 12 per minute. In one of these cases, the disease was of the *Typhoid* type, of a mild grade, not complicated with pneumonitis, convalescence being established on the ninth day after admission. There were no unusual cerebral symptoms in this case. The other case was of *Doubtful type*. In this case, the respirations numbered only 12 the day following an attack approaching to apoplectic coma. Pneumonitis did not exist in this case, and convalescence was established on the ninth day after admission.

In another case, the respirations, on one occasion, were but 7. This diminution in frequency occurred in connection with another modification of the respiratory movements, viz., spasmodic inspiration, which I shall presently consider at some length, and give the concurring circumstances in detail.

*Sighing.* A disposition to sigh is noted in *two* cases, both of the *Typhoid* type. In *one*, it was observed on the first day after admission, and in the other case, after the patient had been in hospital for several days. In both cases, the disease was fatal.

*Spasmodic Inspiration.* The occurrence of an aberration characterized by *spasmodic inspiration*, in other words, the respiration being *shortened* and *quickened*, in several cases, some of which are not contained in this collection, has led me to regard it as an important symptom, significant of a morbid condition seated at the medulla oblongata, compromising that instinctive faculty pertaining to this portion of the nervous centre, upon which the respiratory movements are dependent, and foreboding a fatal termination by apnœa in some instances when other indications of immediate death are not appreciable. I am therefore desirous of studying it with some particularity in the histories in which it is noted. I find that this symptom was present in *eleven* cases, to wit, in *five* of *Typhoid*, *four* of *Typhus*, and in *two* of *Doubtful type*. I will examine the circumstances connected with it in these cases severally.

*Typhoid.* Case 1. In this case the inspiration became notably spasmodic (catching) on the *sixth* day after admission. Before this, no symptoms had occurred affecting very unfavorably the prognosis. The nervous symptoms were somewhat predominant, consisting of considerable delirium at night, somnolency at times during the day, and some subsultus. There had been moderate diarrhœa, and the febrile movement was not greater than in the average of cases.

The following is the record on the *fifth* day after admission:

"Reports much better. Says he slept well. Face and hands still congested. Conjunctiva much injected. Mind dull. Indifferent. Respirations 16, with dilation of alæ nasi. Pulse 108, tolerably developed. Tongue dry and smooth, coated at centre, and moist at sides. Skin rather hot and dry. Great thirst. No pain. Decubitus lateralis et dorsalis. Disposed to somnolency. Delirium during night manifested by incoherent talking and attempting to get out of bed. Two dejections yesterday, and one during night."

This group of symptoms would hardly be considered to denote much danger of a speedily fatal issue. On the next day the following is the record made by me: "Reports better. Delirium during night, as before. Sleeping when I approached the bed, and groaning with each expiration. Readily roused. Protrudes his tongue without difficulty, which is dry on the superior surface, and cracked. Relapses into a somnolent state directly after being roused. Two dejections yesterday, and one in night, the latter involuntary, in bed. Respirations 28, *inspiration shortened and quickened. Occasional stertor in inspiration.* Dilation of alæ nasi. *Slight tracheal rattle.* Slight epistaxis this morning. Pulse 120, moderately developed. Skin warm and dry. *Swallows without difficulty.* Face presents deep congestive redness. Some muscular tremor of upper extremities, and picking at bed clothes observed this morning, none during examination, nor subsultus. Abdomen tender on pressure."

In the above group of symptoms nothing appears foreshadowing a speedily fatal issue, save those referable to the pulmonary system.

Death took place at 11, P. M., of the same day, at which time the following record was made: "This patient continued to fail during the day, the respiration becoming more labored and difficult, and each expiration accompanied by a loud groan. He experienced some difficulty of deglutition a short time before dissolution, but readily swallowed remedies, and nutriment, up to that time. The circulation gradually became enfeebled, but was appreciable at the wrist a few moments before death."

There was no autopsy in this case. The patient suddenly died of apnœa, which was foreshadowed by the symptom under consideration.

Case 2. The inspiration became *spasmodic* on the *seventh* day after admission, the patient not having taken to the bed prior to entering the hospital. The symptoms generally did not denote a severe grade of disease. On the day when the aberration of respiration was noted, the following was the record: "Reports the same. Says he did not sleep well. Two dejections yesterday, and two during the night. Face and upper extremities still congested. Pulse, 92, tolerably developed. Respirations 20, slight dilation of *alæ nasi. Inspiration shortened and quickened.* Slight tremor of muscles of face. Abdomen tympanitic. Tenderness on pressure over right iliac region. No eruption. Some cough. Some appetite. He is quite thirsty."

On the *eighth* day the following is the record: "Reports better. Says he slept well. Some cough, dry. Three dejections yesterday, two in night, and one this morning. Skin warm and mellow. Tongue dry and thinly coated. Pulse 104, soft. *Respirations* 32, *with dilation of alæ nasi, and inspiration*

*short and quick.* Moderate tympanites. Indistinct eruption. Obscure abdominal tenderness. Face and hands much congested. *Slight tremor of muscles of the face when he speaks.* No subsultus."

On the *ninth* and *tenth* days, the symptom under consideration is not mentioned. On the latter date, a slight crepitant rale was discovered, but, in general, the symptoms denoted nothing unfavorable. On the *eleventh* day, the record is as follows: "Reports about the same. Says he slept well. Had two dejections yesterday, two during night, and one this morning. Aspect improved. Less congestion of face and hands. Complains of deafness, and of being tired of the bed. Skin warm and mellow. Tongue moist and clean. Pulse 104, and tolerably developed. Respirations 24, with some dilation of the *alæ nasi.* Some cough and expectoration. Crepitant and bronchial rales intermingled on the right side, posteriorly, middle third of chest."

On the *twelfth* day, the following record was made by me: "Aspect improved, and he reports better. Last evening there occurred a paroxysm of labored respiration, with some lividity of face, tremulousness of hands, and dropping of the lower jaw. A blister was applied to the nucha, and stimulants given more freely. The paroxysm continued about an hour. Afterward, he became comfortable, and passed a good night. Less congestive redness this morning. Respirations 20. *Inspiration shortened and quickened.* Tongue moist and thinly coated. No dejections during day yesterday, two in night, and one this morning. Pulse 110, tolerably developed. Skin warm and moist. Perspired freely this morning, drops of perspiration on face. No delirium, seems perfectly rational. Cough is troublesome, expectoration small, characters not observed. The right chest anteriorly slightly dull. Sibilant rale over both sides anteriorly. Posteriorly, flatness over the left chest, with faint, crepitant rale.

After this date nothing occurred worthy of note, and convalescence was declared on the nineteenth day.

Case 4. In this case, perforation of intestine occurred on the seventeenth day after admission. Prior to this event, the career of the disease was marked by nothing special, save the development of pneumonitis. The aberration of respiration under consideration was noted the day after the perforation, death taking place at evening of the same day. In this instance the symptom had no special significance, inasmuch as the other symptoms associated with the perforation denoted a fatal issue; and, in fact, it was only an element of the moribund state.

Case 5. The symptom in this case is noted in an evening record on the day the patient was admitted. She had been ill five days before entering the hospital. In the morning record the respirations were 28, the pulse 136. Moderate tympanites and tenderness. No cough, etc. At evening, she was quite somnolent, immediately relapsing into a profound slumber after being aroused. *The respirations numbered 7. Inspiration short and quick.* No dilation. Reports that she feels better than in the morning. Has had several dejections, (four or five.) Pulse 144, tolerably developed. No pain in head. Skin warm and dry. Some thirst. Tongue dry and thinly coated. Abdomen tympanitic. No tenderness.

On the following day, the respirations were 24, *inspiration short and quick.* Pulse 140. Delirious during night. Considerable tympanites, and tenderness in iliac regions, etc.

On the next day, the symptom under consideration is not stated. Pulse 136, etc. Half an ounce of brandy, hourly, was administered. During the day, and at evening, the pulse was 116, and all the symptoms denoted improvement. Convalescence was established in a few days afterward.

In this instance, the symptom was associated with other symptoms which rendered the prognosis unfavorable, but, happily, a change for the better occurred, and the patient recovered.

*Typhus.* Case 1. The inspiration became notably spasmodic, in this case, on the *fifth* day of the admission, the patient not having taken to the bed prior to entering the hospital. The symptoms on the *fourth* day, as recorded by me, are as follows: "Reports the same. Aspect bright. Face deeply congested. Mind seems clear, but he was delirious during the night. Sordes on teeth. Tongue moist, and thinly coated. Two dejections yesterday. Complains of thirst. Skin dry and rather hot. Pulse 120, soft and feeble. Complains of cough, and says the act of coughing occasions pain in the belly. Respirations 24. Dilation of *alæ nasi.* Complains if firm pressure is made over the abdomen. Slight meteorism. Eruption very copious, almost confluent, over the abdomen, extends to the upper and lower extremities, even to back of hands."

The next day as follows: "Aspect same. Incoherency during night. Urinated in bed. Tongue coated, and somewhat dry in the centre; readily protruded. One dejection this morning. Respirations 24. *Inspiration shortened and quickened.* Dilation of *alæ nasi.* Cough continues. Congestive redness diminished. No deafness apparent. No epistaxis. No abdominal distention, nor tenderness. Skin warm and dry. Pulse 120, small and feeble. In this group of symptoms, nothing certainly denotes an unusual gravity of disease, or impending danger, excepting the modified respiratory movements. The significance attached to the symptom at the time, is indicated by the fact that a blister to the nucha was added to the treatment.

The next day it is noted that the respirations were *still catching*, numbering 36; pulse 120; *no physical signs of consolidation of lungs;* delirious during night, etc.

The next day respirations 40, *still catching;* delirious during night; pulse 134.

Next day aspect much worse; *inspiration short and catching; expiration attended by a groan;* expression vacant; endeavors ineffectually to reply to questions; cannot protrude the tongue; pulse 146. Died at 2, P. M. The autopsy was limited to the abdomen.

Case 2. The inspirations were observed to be *slightly* spasmodic, in this case, on the *sixth* day. Previously to this time, the symptoms had not denoted unusual gravity of disease. The symptoms associated on the sixth day, were the following: "Aspect improved; pulse 120; skin warm and dry; tongue moist and thinly coated; two dejections; respirations 32, *and slightly catching*."

On the following day, the patient reported pretty well. Aspect about the same. Tongue clean, dry, and somewhat fissured. No dejections. Respirations 40, *inspiration somewhat shortened and quickened.* Pulse 128, small and feeble. Skin cool and dry. Deafness continues. No abdominal tenderness. Talks incoherently. A blister to the nape of the neck is embraced in the directions for treatment on this day.

On the next day, "Aspect improved. No manifestations of delirium. Tongue clean and somewhat dry. One dejection. Abdomen soft, without tenderness. Pulse 120. Respirations 36, *still catching.* Complains of deafness."

I recollect being informed, on this day, before I entered the ward, that the patient was better, and, in fact, all the symptoms, irrespective of the respiration, gave evidence of improvement. The respirations, too, were diminished in frequency.

Death took place before the following morning. The details, after the morning record just quoted, are not given, nor the hour at which the death of the patient occurred. There was no autopsy in this case.

Case 3. In the history of this case, it is noted, on the *eighth* day, that the *inspirations were somewhat shortened;* respirations numbering 28. He was delirious during the night. Disposed now to somnolency, but readily roused. Pulse 112, and well developed. Eyes somewhat injected. Considerable congestive redness of face. Tongue dry and hard in centre.

Abdomen moderately meteorized. Eruption faded. A blister to nucha was directed, and sinapisms to the calves of legs.

The day following, his aspect was much improved. He was perspiring profusely. Respirations 28. Pulse 96, etc. In two days, convalescence was established.

Case 4. The *inspirations became somewhat spasmodic* on the eleventh day. The patient was a candidate for the sisterhood, and was attacked with the disease in the hospital, having contracted it, doubtless, by contagion. Prior to the occurrence of this symptom, nothing appeared to denote unusual gravity of disease; and on the eleventh day, excepting the respiration, the symptoms were not unfavorable. The following is a transcript of the record on this day: "Passed a comfortable night. Less delirium. Bowels moved once yesterday. She reports better. Tongue thinly coated, and dry in centre. Sordes on teeth. Skin warm and slightly moist. Pulse 120. Respirations 24. *Inspirations somewhat shortened and quickened.* No epistaxis. She has slight cough. Abdomen considerably meteorized."

On the next day, the record, made by me, reads as follows: "Last evening this case assumed an alarming aspect. The rhythm of the respirations became affected to a greater extent than in the morning — the *inspirations much shortened and quickened.* It was difficult to arouse her. The abdomen was much meteorized. A blister to the neck was directed; an enema, and turpentine fomentations to the abdomen. This morning there is a marked improvement. Respirations 30, and rhythm quite normal. Abdomen moderately meteorized, and soft. Pulse 100. Skin warm and moist. Easily roused, and appears rational.

On the day following, the inspiration became again, in some degree, similarly affected. The respirations numbered 40. Pulse 120. Cough became a troublesome symptom on this day, and physical exploration disclosed the occurrence of pneumonitis. On the next day, the symptoms indicated improvement. Pulse 104. Respirations 40, and rhythm normal.

Convalescence was shortly established.

*Doubtful Type.* Case 1. The symptom under consideration occurred, in this case, on the second day after admission. In the morning of the day on which it occurred, the following record was made: — "Reports better. Says he slept well. Face and hands moderately congested. Skin warm and dry. Tongue dry and coated, dark. Says he has some appetite. Great thirst. No dejection. No abdominal tenderness. No eruption. Pulse 104, soft, tolerably developed. Says he has no cough. Respirations 16, without dilation. He was delirious during night, manifested by incoheren talking. Some picking at bed clothes this morning. He is quite restless, frequently changing his position in bed."

At six, P. M., of the same day, the following record was made: — "Copious perspiration. Hands cold and clammy. Tongue dry and fissured. Pulse 140, small, and feeble. *Respiration irregular, catching.*"

In the morning record of the succeeding day, which was made by me, it is stated: — "The symptoms in this case, last evening, denoted a striking change for the worse, and the patient was supposed to be nearly moribund. Stimulants were freely administered, sinapisms applied to the extremities, a blister to the nucha, and snow to the head. He began to improve at about ten, P. M., and during the night, gradually became more comfortable.

On the following day he "Reports pretty well. Says he slept well. Face and upper extremities moderately congested. Skin warm and moist. Some meteorism. No abdominal tenderness. Pulse 68.

Convalescence was established in a few days.

Case 2. In this case the disease was of a severe grade, and complicated with pneumonitis. On the *twelfth* day after admission, the following were the symptoms: — Aspect the same. No manifestations of delirium. Circumscribed flush of cheeks. Skin moist. Pulse 108. Respirations 40, rhythm normal. Considerable tympanites.

On the *thirteenth* day, reported pretty well. Aspect same. Pulse 112. Skin perspiring, cool. Respirations 52. *Inspiration somewhat shortened and quickened.* Cough diminished. Two dejections, etc.

On the *fourteenth* day, the respirations were 60, but rhythmical. Pulse 100, etc.

This patient recovered without farther untoward symptoms.

The foregoing are all the instances in which any reference is made, in the histories, to the symptom under consideration. Had it occurred in other cases, it would not have been likely to have escaped notice, for I was specially interested in observing it during the time these cases were collected. It may, however, have been present in some other cases in the last moments of life, without being noted, forming, as it probably does, in many cases, an element of the mode of dying by apnœa.

I have given the circumstances connected with this symptom in detail, with a view to exhibit all the facts contained in the present collection of cases bearing upon its significance and value, as a symptom, occurring occasionally during the career of Continued Fever. These facts appear to show that, although sometimes observed without being followed by symptoms denoting great gravity and danger, it is generally either a forerunner of, or associated with a morbid condition of the nervous system eventuating in, or tending to coma, and death by suspension of that faculty of the nervous system which presides over the respiratory function. It is a symptom of ominous import, which may be present, not only when the associated symptoms do not give any occasion for alarm, but even when the latter would signify diminished severity of disease. The details that have been given seem to establish these conclusions, which invest the symptom with considerable importance in prognosis, and as a means of anticipating an event, to avert which, therapeutical measures may be seasonably, and perhaps successfully employed. The significance of this symptom was forcibly impressed on my mind by a case in private practice, that came under observation some weeks since, of which I did not keep notes. I was requested to visit a young girl, a domestic, who had been ill several days without medical advice. I found the patient evidently laboring under Continued Fever, the type being, at that time, indeterminate. There was high febrile movement — frequent pulse, hot skin, etc., but nothing to denote immediate danger, save that the inspirations were somewhat spasmodic. In the afternoon the symptoms generally, and that just mentioned, remained the same; but I hesitated to believe that the latter was so ominous as I had been led to suppose from cases before observed. I encouraged the suspicion, in my own reflections, that I might have overlooked its occurrence in many cases in which no sudden change for the worse had followed, and, thus, I resolved not to anticipate any such change. During the night I was summoned in haste to this patient, and I immediately surmised what I discovered when I reached the house. Nothing had occurred during the evening to alarm the family, who were, of course not cognizant of the train of reflection I had indulged, but, at length, the noisy respiration of the patient attracted attention. She could with great difficulty be roused, and soon this was impossible. Apoplectic coma had in fact supervened, and death occurred in three or four hours after the discovery was made that she was in a dangerous condition.

On the pathology or proximate cause of the nervous condition which this symptom sometimes foreshadows, some remarks have already been submitted in a more appropriate connection, viz., in the section on the symptoms referable to the nervous system, under the head of apoplectic coma.

The peculiar modification of the respiration which I have here described as consisting in a spasmodic, or shortened and quickened inspiration, does not appear to have excited that attention which its importance, as a symptom, claims. I suppose it is to this modification that Prof. Bartlett refers, in his able treatise on fever, in speaking of an *irregular* and noisy respiration which he distinguishes as *cerebral.* Disturbance of the respiration, not dependent on the condition of the lungs, is mentioned by Dr. Nathan Smith' and by Chomel, of France, as a symptom denoting great danger.

*Dilation of the Alæ Nasi.* More attention was paid to this symptom in recording the histories of the cases in the present collection, than of those before analyzed. Its presence was noted in *eleven* cases of *Typhoid,* and in *five* cases of *Typhus.* Its absence was noted in *eight* cases of *Typhoid;* in the remainder of the cases in both types in which it is not stated to have been present, nothing is said respecting it. In *fifteen* of the *sixteen* cases in which it was observed, the respirations were, at the same time, more or less increased in frequency. In the single case in which the respirations were not accelerated, the dilation was slight, and is only noted to have been present on one day. In *five* of the cases in which it was observed, the fever was complicated with pneumonitis. *Six* of the cases were characterized by the occurrence of a quickened and shortened inspiration. In other words, *six* of *nine* cases thus characterized, presented this symptom.

*Nine* of the cases proved fatal. In other words the symptom was present in *nine* of *eleven* fatal cases, and in the *two* in which it is not stated to have been present, nothing is recorded in the histories respecting it.

These facts suffice to show that dilation of the alæ nasi is to be considered almost uniformly a sign of disorder of the respiratory movements incident to affections which, like pneumonitis, compromise the pulmonary organs, or to functional aberrations dependent on ulterior morbid conditions. It is not, however, true, on the other hand, that disorder of the respiratory movements is as uniformly accompanied by this sign; for, in several of the cases in which it was noted that the alæ did not dilate, the respirations were accelerated, in some instances constituting a prominent symptom. In *one* case, at least, in which the inspirations were quickened and shortened, the alæ did not dilate. In none of the cases in which pneumonitis complicated, is it noted that dilation was not present, nothing being stated with respect to this point in the cases in which the presence of this symptom is not affirmed.

*Epistaxis.* The occurrence of this symptom is noted in the histories of *nine Typhoid* cases. Of the remaining *nineteen* cases, nothing is stated relative to the symptoms in *eleven.* It is stated that epistaxis did not occur in *six;* and in *three* cases sputa expectorated were observed to be streaked with blood, which may have been derived from the posterior nares, but this is not certain. This enumeration does not, it will be borne in mind, embrace the access, or forming stage of the diseases. Attention was directed certainly in most, if not all the cases, to this symptom, and in those, in the histories, of which nothing is stated on the subject, it may be presumed to have been not present. It may have occurred, in some instances, prior to the patients coming under observation, and not be embraced in the records of the previous history, although inquiries were generally made respecting this point.

The proportion of *Typhoid* cases in which epistaxis occurred is somewhat greater in the present collection, than in that previously analyzed, the numerical relation being as 19 — 29, is to 8 — 30 or 4 — 15.

The periods in the disease, dating from the time of admission, when the epistaxis occurred, were as follows, in the cases respectively: — Case 1, third day; case 2, sixth day (last day of life); case 3, second day; case 4, first day; case 5, sixth, seventh, and eighth days; case 6, first and second days; case 7, seventh day; case 8, on several days, dates not given; case 9, third day. This symptom, thus, observes no law of uniformity as respects the time of its appearance.

The hæmorrhage was either slight, or quite moderate, in all but *two* cases. In *one* of these cases the epistaxis occurred on the sixth, seventh, and eighth days, and was considerable in degree. In the other case it occurred on the first and second days, and was so profuse that the propriety of plugging the nostrils posteriorly was considered, although not found to be necessary. Aside from these two cases, it is not noted that the epistaxis occurred more than once in but a single case.

Of the cases of *Typhus* the occurrence of epistaxis is noted in *none;* nor were sputa streaked with blood observed in any case. In the first collection epistaxis was noted in *two* of *thirteen* cases of the *Typhus* type.

In the *nine* cases of *Doubtful type*, epistaxis occurred on the second and fourth days, in *one* case; and on the first and second days in *one* case. In *three* cases it is stated that this symptom was not observed, and of the *five* remaining cases the histories do not contain any statements with regard to it.

It is perhaps worthy of notice, that none of the cases in which epistaxis occurred, proved fatal. This is the more interesting from the fact that the same was true of the *Typhoid* cases in the first collection. It would thus seem that this symptom is not to be considered any evidence of unusual gravity in the disease.

(To be Continued.)

